# Reproductive Interference in an Introduced Bumblebee: Polyandry may Mitigate Negative Reproductive Impact

**DOI:** 10.3390/insects10020059

**Published:** 2019-02-22

**Authors:** Koji Tsuchida, Ayumi Yamaguchi, Yuya Kanbe, Koichi Goka

**Affiliations:** 1 Laboratory of Insect Ecology, Faculty of Applied Biological Sciences, Gifu University, Yanagido 1-1, Gifu 501-1193, Japan; bumblebee12357@gmail.com (A.Y.); Yuya.Kanbe@arysta.com (Y.K.); 2 Arysta Lifescience Corporation Bio Systems, Asia and Life Science Business Group 418-404 Nishihara, Tsukuba, Ibaraki 305-0832, Japan; 3 National Institute for Environmental Studies, Onogawa 16-2, Tsukuba, Ibaraki 305-0053, Japan; goka@nies.go.jp

**Keywords:** reproductive interference, bumblebees, hybrid production, hybrid inviability

## Abstract

As a signature of reproductive interference (RI), we reviewed hybrid production in eusocial bumblebees in Japan, by comparing introduced *Bombus terrestris* with native *B. ignitus* in Honshu (main island of Japan) and with native *B. hypocrita sapporoensis* in Hokkaido (northern island of Japan). In this review, we present additional new data showing hybrid production between introduced *B. terrestris* and native *B. ignitus* in Honshu. Interspecific mating with introduced *B. terrestris* disrupts the reproduction of native *B. h. sapporoensis* and *B. ignitus*, which belong to the same subgenus of *Bombus*, through inviable egg production. This interference appears to facilitate species replacement on Hokkaido. Simultaneously, the mating frequencies for queens of *B. terrestris* have increased, suggesting that polyandry might evolve in response to the extent of RI between *B. terrestris* and *B. h. sapporoensis*. To suppress the population size of *B. terrestris* in Hokkaido, two methods have been proposed: the mass release of *B. h. sapporoensis* males to induce RI between the two species and the spraying of insecticides against foraging workers so that the workers will carry the insecticides back to their colonies, killing the immature bees within the colonies. A candidate insecticide type is insect growth regulator, which may disrupt larval development without any apparent effect on foraging workers.

## 1. Introduction

Negative interspecific interactions between closely related species during the process of mating can lead to species exclusion [1,2,3,4,5,6]. Theoretically, such sexual interactions, known as reproductive interference (RI), have been suggested to protect the coexistence of the two interacting species, leading to parapatry [1,2]. 

RI consists of several sequential steps leading to copulation, where copulation between different subspecies or species is the final step. Hybrid inviability and/or infertility is more likely to have negative impacts on the less abundant subspecies or species within the pair. As a result, the rarer subspecies or species is likely to decline in abundance or even become extinct. For example, during courtship, one whitefly biotype was observed to disturb the courtship behaviour of another biotype and biotype replacement occurred thereafter [3]. Similarly, the presence of some biotypes can affect the reproduction of other biotypes in a manner similar to a biased sex ratio [7]. Thus, RI was proposed as a promising method to eradicate the invasive melon fly using the sterile insect technique via γ-irradiation [8] and the mass release of males infected with *Wolbachia* strains to control wild mosquitos [9].

In the eusocial Hymenoptera, few reports exist on courtship behaviours between pairs of subspecies or species, partially because such research topics lie outside the focus of mainstream studies. In particular, to our knowledge only a few reports have described RI in eusocial insects [10,11,12,13]. Eusocial insects have a colonial lifestyle, in which the queen(s) monopolize(s) colony reproduction and workers forfeit their own reproduction. Colonies vary in the number of queens, showing either monogyny or polygyny. Variation also exists in the mating frequency of the queen, showing either monandry or polyandry. These variations in reproductive traits could complicate the detection of hybrids, particularly under conditions of polygyny and polyandry. However, the mechanism by which RI could cause eradication or extinction of one species of a closely related eusocial insect species pair remains poorly understood. We herein review hybrid production as a characteristic of RI in eusocial bumblebees in Japan, present new data showing hybrid production between introduced *Bombus terrestris* and native *Bombus ignitus* on Honshu (main island of Japan) and describe recent developments around invasive *B. terrestris* in Japan since our last review [14]. We also discuss the extent to which RI may affect the eusocial traits of this group. We propose specific control methods for invasive *B. terrestris* on Hokkaido (northern island of Japan). 

## 2. Bumblebees in Japan

**Figure 1:**
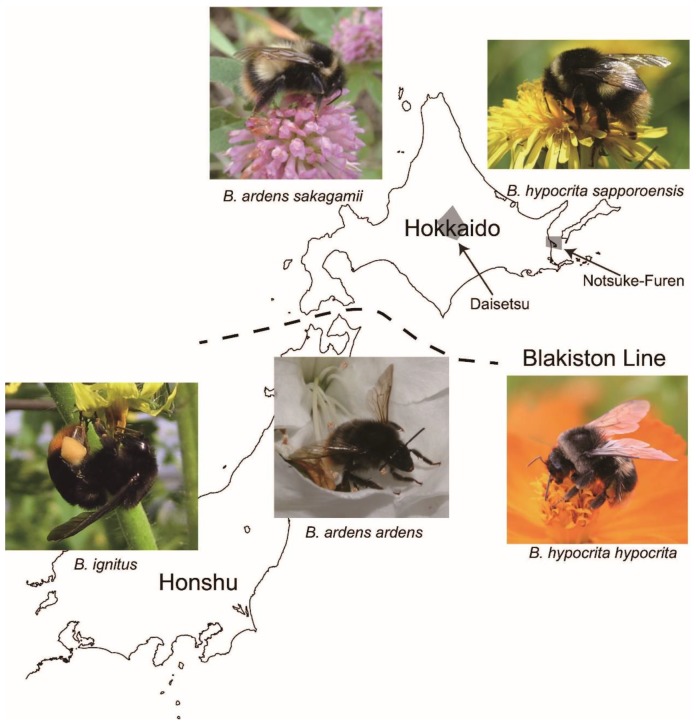
Distributions of representative native bumblebees on Hokkaido (northern island of Japan) and Honshu (main island of Japan). The Blakiston Line between Hokkaido and Honshu divides the fauna of each island (photos by M. Inoue and Y. Kanbe). The two shaded areas are Daisetsu National Park and Notsuke-Furen Natural Park.

In bumblebees, only fertilized queens overwinter and they initiate colonies in spring. After months with only a queen and workers, in late summer a bumblebee colony produces new queens and males for reproduction. After mating, spermatozoa are stored in the queen’s spermatheca until spring, when she starts a new nest and eggs are fertilized. Within their native range, *Pyrobombus* species are slightly polyandrous and *Bombus* species, including *B. terrestris*, are monandrous (Table S1) [15,16,17,18,19,20,21,22]. Japan has at least 22 species of native bumblebees including *B. hypocrita* and *B. ignites*, in the same subgenus (*Bombus*) as *B. terrestris* (Figure 1), which is generally a monandrous subgenus [23]. 

Bumblebees are important pollinators of wild flora, as well as agricultural crops. Among bumblebee species, *B. terrestris* is an effective commercial pollinator used in greenhouses worldwide. Pollination by *B. terrestris* reduces the need for hand pollination and chemical hormones, which leads to reduced use of insecticides [24]. Therefore, in addition to the natural distribution of *B. terrestris* over continental Europe from west of the Urals to the Near East and North Africa, this species is now spreading through several countries in the Southern Hemisphere and Asian countries, as an alien species [24].

In contrast to its importance as a pollinator, *B. terrestris* has also been recognized as an invasive species [25,26]. The trade in this species facilitates its establishment in new areas, where it can have ecological impacts on native flora and fauna. Indeed, the deliberate and successful establishment of feral colonies of *B. terrestris* was reported in New Zealand in the early 20th century [26], followed by Tasmania [27], Israel [28] and Japan [29]. In 1991, *B. terrestris* was first introduced to Japan and in 2004, about 70,000 colonies were released into greenhouses throughout Japan [30]. The unrestricted mass release of *B. terrestris* into new environments affected the native fauna and flora after *B. terrestris* escaped from greenhouses. Documentation of wild *B. terrestris* nests in Japan indicates that the species has become naturalized [31,32]. Since the process of naturalization began, the number of *B. terrestris* individuals has steadily increased, particularly in Hokkaido, probably because its climatic conditions are more suitable for *B. terrestris* than those of Honshu.

## 3. Negative Impacts on Native Fauna and Flora

With respect to *B. terrestris* in Japan, we detected four candidate negative ecological impacts on native fauna and flora. First, *B. terrestris* and native species compete for food resources and nesting sites. In Hokkaido, the distributions of *B. terrestris* and native *B. ardens sakagamii* are mutually exclusive, probably due to negative interactions between the two species over resources [33]. In these negative interactions, the legitimate native pollinator *B. a. sakagamii* visited fewer flowers of the native plant *Corydalis ambigua* within inflorescences containing more flowers that were robbed by the alien and non-legitimate pollinator *B. terrestris*. Such nectar robbing without pollen causes a reduction in the frequency of visits by *B. a. sakagamii* to flowers within inflorescences [34]. In Hokkaido, considerable niche overlap is present in flower resource use between *B. terrestris* and native *B. hypocrita sapporoensis*/*B. pseudobaicalensis* [35]. *B. terrestris* may also compete with *B. h. sapporoensis* for nest sites and the large reduction observed in the number of *B. h. sapporoensis* queens indicates that *B. terrestris* may cause local extinction of native bumblebees. Second, parasites of *B. terrestris*, such as the tracheal mite *Locustacarus buchneri*, might be co-introduced and affect native bumblebee species through host switching [36,37]. The invasive microsporidian parasite *Nosema* is likely a cause of the decline in natural bumblebee populations in the USA [38] but quantitative data on the prevalence of *Nosema* in Japan is still lacking. *N. bombi* has been detected from *B. terrestris* but not from *B. h. sapporoensis* in Hokkaido [39]. This finding indicates that spill over of *N. bombi* from greenhouses will be highly likely to occur and the parasites will spread among native species in the near future. Third, mutualism between native plants and native pollinators may be disturbed [34], for example, a decrease in fruit set by the native plant *C. ambigua* is likely caused by non-legitimate visits from *B. terrestris* that reduce legitimate visits from *B. ardens* due to nectar robbing. Fourth, interspecific mating between *B. terrestris* and native species could result in genetic deterioration of the native species [40,41]. 

## 4. Bombus Ignitus in Honshu

Among the four negative impacts outlined above, genetic deterioration due to interspecific mating is the most serious, as humans are unable to exclude and offset such deterioration after genetic amalgamation and sterilization begins. In Honshu, interspecific mating is expected to occur between native *B. ignitus* and introduced *B. terrestris*, because interspecific mating between the two species readily occurs in captivity and they may distribute sympatrically. Therefore, we conducted experimental pairing of the two species according to the procedure used in our previous report on *B. terrestris* × *B. h. sapporoensis* [10]. 

### 4.1. Materials and Methods

We conducted an interspecific mating experiment between *Bombus terrestris* and the Japanese native species *B. ignitus. B. terrestris* queens and males were collected from *B. terrestris* colonies that had been artificially propagated by Api Co. Ltd., (Gifu, Japan). Colonies of *B. terrestris* were reared from mated queens, which were held for 3 months at 2 °C before nest initiation and then introduced into a rearing chamber maintained at 28 °C and 60% relative humidity (RH) with a *B. terrestris* pupa to elicit oviposition and provided with sufficient pollen and sugar solution. Newly emerged virgin queens and males were collected from these colonies and held until copulation in sex-separated cages (30 × 30 × 30 cm) with sufficient pollen and sugar solution. *B. ignitus* queens and males were also collected from *B. ignitus* colonies that had been artificially propagated by Api Co. Ltd. These queens were artificially induced to establish colonies using similar conditions as for *Bt*. Newly emerged *B. ignitus* virgin queens and males were collected upon emergence. For interspecific mating (*B. ignitus* queens × *B. terrestris* males and *B. terrestris* queens × *B. ignitus* males), 20 to 30 queens (6–10 days old) and three times as many males (10–20 days old) were released into cylindrical mating cages made of fine netting (40 cm diameter and 80 cm high). After copulation within three hours after the releasing, the males were collected and stored at −80 °C.

After copulation, the mated queens were induced to hibernate and were stored in a refrigerator at 2 °C for 3 months. Subsequently, the queens were introduced into a rearing chamber (maintained at 28 °C and 60% RH) with a *B. terrestris* pupa to elicit oviposition and provided with sufficient pollen and sugar solution.

The eggs oviposited by *B. ignitus* queens that had copulated with *B. terrestris* males and vice versa, were gently collected with a fine brush on the day of oviposition and placed on a Petri dish (maximum of 10 eggs per dish) lined with a paper towel moistened with tap water. The dishes were sealed with Parafilm and maintained at 30 °C. Every day, several eggs were collected from the dishes and placed into 10% formaldehyde for histological study of their development. They were then individually embedded in wax; serial sections were cut, stained with haematoxylin-basic fuchsin and methylene blue-alcoholic eosin and mounted in balsam. The remaining eggs were checked daily under a dissecting microscope. Hatched larvae were collected and stored at −80 °C for genotyping. If eggs remained unhatched after day 6, we regarded them as inviable and the experiment ended on day 7 after oviposition. When eggs were viable, larvae hatched 5–6 days after oviposition. As a control, the same procedure was used for intraspecific mating. We excluded data on eggs used for histological study from our calculation of egg viability from inter- and intraspecific mating.

We genotyped eggs and hatched larvae using the microsatellite loci B11, B100, B124, B126 and B132 for colonies derived from a *B. ignitus* queen × *B. terrestris* male and vice versa, as described by [15]. PCR amplification (10 µL total volume) was carried out using 1 µL of diluted genomic DNA (≈ 1 ng), 1 µL of primer mix (2.5 µM), 0.1 µL of 10 mM dNTP mix, 1 µL of 10× buffer (with *Taq*, 1.5 mM final concentration) and 6.85 µL of dH_2_O. We varied the concentration of MgCl_2_ according to the PCR conditions for each locus. PCR was carried out using a thermal cycler (TP-240; TAKARA, Ohtsu, Japan). The PCR products were electrophoresed on 8~10% native acrylamide gels and visualized using silver staining [42]. Genotyping was conducted using 1D Image Analysis Software (Kodak, New York, NY, USA) installed on a Macintosh computer. Frequency data were compared using Fisher’s exact probability test with the sequential Bonferroni correction (α = 0.05).

### 4.2. Results and Discussion

We introduced 37 *B. ignitus* queens into our mating cage, of which 26 (70.2%) copulated with *B. terrestris* males. We could not measure the corresponding frequency for *B. terrestris* queens × *B. ignitus* males due to the small sample size

*Bombus ignitus* queens that had copulated with *B. terrestris* males were induced to hibernate and 49 *B. ignitus* queens successfully began oviposition. In total, 566 eggs were collected. Similarly, *B. terrestris* queens that had copulated with *B. ignitus* males were induced to hibernate; however, only one queen successfully began oviposition and 13 eggs were collected. The hatching rate of eggs derived from *B. ignitus* queen × *B. terrestris* male mating was 3.9% (22/566), which was significantly lower than those of intraspecific mating in *B. ignitus* and *B. terrestris* (Table 1; Fisher’s exact probability test with sequential Bonferroni correction). 

Values followed by the same letters are not significantly different based on Fisher’s exact probability test with sequential Bonferroni correction (α = 0.05). Unfortunately, we could not obtain credible data for the corresponding hatching rate for *B. terrestris* queen × *B. ignitus* male mating due to the small sample size.

We genotyped 28 eggs and 15 larvae derived from *B. ignitus* queen × *B. terrestris* male mating and vice versa using microsatellite markers (Table 2). The zymograms of 24 of the 28 eggs showed two-band patterns at one or more loci and agreed with the expected diploid genotypes. The remaining four eggs had one band at each locus and agreed with the expected haploid genotypes. The genotypes of the 15 larvae had one-band patterns and agreed with the expected haploid genotypes. Similarly, we genotyped five eggs and four larvae derived from *B. terrestris* queen × *B. ignitus* male mating. The zymograms revealed that one of the five eggs and all four larvae were haploid.

**Figure 2:**
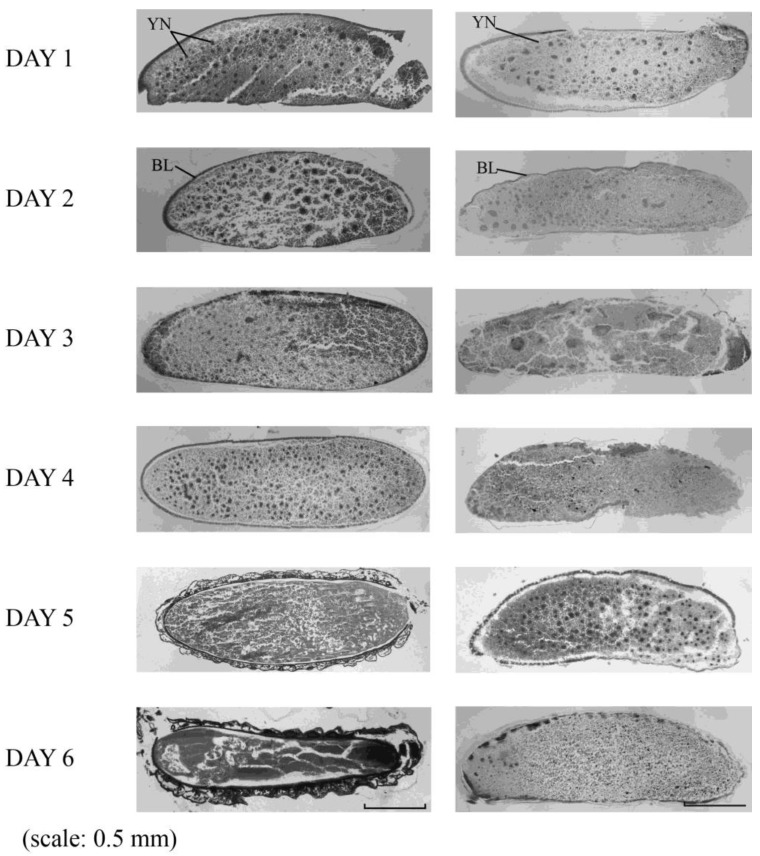
Serial sections of eggs 1 to 6 days after oviposition from interspecific mating between a *Bombus ignitus* queen and a *B. terrestris* male under laboratory conditions. The left column is a series of pictures of intraspecific mating; the right column includes pictures of interspecific mating. YN, yolk nucleus; BL, blastoderm. Scale: 0.5 mm.

Figure 2 shows serial histological sections of 1–6-day-old eggs derived from intraspecific *B. ignitus* mating and interspecific *B. ignitus* queen × *B. terrestris* male mating. The sample of serial histological sections comprised 42 intraspecific *B. terrestris* matings and 47 interspecific *B. ignitus* queen × *B. terrestris* male matings. In eggs originating from intraspecific mating, yolk nuclei (YN) were present 1 day after oviposition and blastoderms (BL) formed on day 2. The larval shape was reached on day 6. By contrast, serial sections of 47 eggs derived from interspecific mating showed that, although YN and BL were present on days 1 and 2, respectively, there were no larvae shapes on day 6.

Our study revealed that under laboratory conditions, *B. ignitus* queen × *B. terrestris* male matings produce hybrid diploid eggs. However, no diploid larvae hatched from the colonies derived from queens that mated inter-specifically. Histological studies of eggs derived from interspecific mating revealed that the development of these hybrid eggs had terminated and they were inviable. Therefore, it is unlikely that viable diploid egg production occurs in crosses of *B. ignitus* and *B. terrestris*, as observed in *B. terrestris* and *B. h. sapporoensis* [10], due to strong post-zygotic reproductive isolation mechanisms. However, hybrid female production has been reported from *B. terrestris* queen × *B. h. sapporoensis* male mating in Japan [40] and similar hybrid female production from *B. ignitus* queen × *B. terrestris* male mating was reported in Korea [41]. These results suggest that bumblebees may have the ability to reproduce through interspecific hybridization or thelytokous parthenogenesis. Unfortunately, neither of these reports confirmed the genetic background of the hybrid female. The frequency of hybrid production is low and hybrid female production from *B. terrestris* queen × *B. ignitus* male mating was not observed [41]. Therefore, although further studies using genetic tools are needed to confirm hybrid female production, the frequency of hybrid production appears to be minimal at present in Japan. One-fifth of *B. hypocrita hypocrita* queens in northern Honshu contained spermatozoa from *B. terrestris* males (Table 3), suggesting that interspecific mating between *B. ignitus* and *B. terrestris* is possible in the field, as well as under laboratory conditions. Further studies are needed to evaluate the frequency of interspecific mating between *B. ignitus* and *B. terrestris* in the field in Honshu, where the population of *B. terrestris* will likely increase in the near future.

## 5. Bombus Hypocrita sapporoensis in Hokkaido

In a previous study [10], we conducted artificial interspecific mating between *B. terrestris* and *B. h. sapporoensis* and the resulting mated queens were artificially induced to found colonies. Eggs that were oviposited from *B. terrestris* queen × *B. h. sapporoensis* male mating and vice versa, were checked for their sex and larval development. Although eggs derived from intraspecific mating within *B. terrestris* and *B. h. sapporoensis* hatched more than 80% of the time, eggs derived from interspecific mating between *B. terrestris* and *B. h. sapporoensis* hatched less than 10% of the time. Although the majority of eggs derived from *B. terrestris* queen × *B. h. sapporoensis* male mating were diploid, all of the hatched larvae derived from this pairing were haploid, showing that diploid eggs could not hatch normally and this result was confirmed through histological study.

The frequency of interspecific mating between *B. terrestris* and native Japanese bumblebees was estimated using DNA extracted from spermathecae of collected native queens (Table 3). The long-wavelength rhodopsin gene sequences indicated that 30.2% of the examined spermatozoa stored in queens of *B. h. sapporoensis* on Hokkaido contained spermatozoa from *B. terrestris* males [11]. 

The reason for such frequent interspecific mating in the field is likely due to the formerly allopatric distribution of the pairs of species, leading to a lack of prezygotic barriers to reproduction because reinforcement would not have had the opportunity to evolve [43]. In bumblebees, both male and female sex pheromones are important for copulation. In *B. terrestris*, female- and male-produced compounds act as sex pheromones and males patrol their flight routes, along which they deposit species-specific scent marks to attract virgin queens [44]. After a virgin queen has landed in the patrol area of a male, the female sex pheromone is released to stimulate the mating behaviour of males in close proximity [45]. Interspecific activity of female sex pheromones is present in *B. ignitus* and *B. h. hypocrita*, whereas it is absent for male sex pheromones of these two sympatric species [46]. In general, differences in sex pheromones are associated with species divergence. For instance, populations of the European bumblebee *B. lapidarius* were isolated in several allopatric glacial refugia and likely diversified in terms of the chemical composition of their male sex pheromones according to each populations’ phylogeographic history [47]. Bumblebees discriminate among conspecific mating partners via male sex pheromones but the species discrimination ability of virgin queens and males is disrupted at close range.

Another factor that could explain the high frequency of interspecific mating is that *B. terrestris* males are predominant in the pool of males near greenhouses. As noted above, a substantial number of *B. terrestris* males have already escaped from greenhouses on Hokkaido, with *B. terrestris* males outnumbering *B. h. sapporoensis* males by a factor of more than 50 during the autumn mating season [35]. Both the ambiguity of species discrimination and the numerical predominance of *B. terrestris* males in the male pool could be responsible for the high frequency of interspecific mating. Until the introduction of *B. terrestris*, allopatric distribution alone was sufficient to prevent interspecific mating between *B. terrestris* and *B. h. hypocrita* or *B. h. sapporoensis*. This finding is a good example of an introduced alien species easily breaking down a reproductive barrier that was constructed through a long evolutionary history. 

Together, these findings indicate that RI by *B. terrestris* disrupts the reproduction of *B. h. hypocrita*, *B. h. sapporoensis* and *B. ignitus*, which all belong to the subgenus *Bombus*. Similar RI has been observed in *Apis mellifera* and *Apis cerana* pairs [12,13]. In addition to RI against native *B. hypocrita* queens, the continuous release of *B. terrestris* queens and males from greenhouses will also promote the rapid growth and spread of this alien species in Japan. In response to numerous studies and after 15 years of unregulated use, the Invasive Alien Species Act of the Japanese Ministry of the Environment finally classified *B. terrestris* as an invasive alien species and prohibited the import and local production of colonies without certification [14]. Since the enactment of that law in 2006, all approved populations of *B. terrestris* must be kept in enclosed places, such as greenhouses with nets, from which *B. terrestris* cannot escape. Furthermore, approved users are obligated to incinerate their used hives, which can potentially produce males and new queens. In addition, the commercialized *B. ignitus* is recommended for use on Honshu. 

Although RI also likely disturbs *B. terrestris* reproduction due to native Japanese species, *B. terrestris* population densities have appeared not to decline, particularly in lowland areas of Hokkaido. This unexpected result is worth further study. RI might be asymmetrical, such that queens of native species suffer more negative effects from males of *B. terrestris*, due to less successful species recognition by native species. This hypothesis would be interesting to test in the field.

## 6. Reproductive Interference and Polyandry Evolution

Although there have been no reports of substantial numbers of naturalized colonies of *B. terrestris* on Honshu since the enactment of the law (Invasive Alien Species Act), if our estimate of the frequency of interspecific mating between a queen of *B. h. hypocrita or B. h. sapporoensis and B. terrestris* male continues in future years, the frequency (≈ 30%) [11] could become less than 0.1% by the 6th year (0.3^6^ ≈ 0.07%). This suggests that both native sub-species will eventually go extinct due to RI through interspecific mating, particularly on Hokkaido. Indeed, a dramatic change in species composition was observed over three seasons in Hokkaido in areas close to greenhouses; in spring 2003, 16.51 queens/hour were observed, of which 46.9% were *B. terrestris* and 53.1% were *B. h. sapporoensis*, while in spring 2005, the frequency was 15.37 queens/hour, of which 99.6% were *B. terrestris* and 0.4% were *B. h. sapporoensis* [35].

When RI occurs, polyandry should be adaptive because it can provide more opportunities to produce viable offspring than monandry. However, the data available at present indicate that most bumblebee species are monandrous and even known polyandrous species mate with a frequency close to one, aside from *Pyrobombus* (Table S1). If native *B. h. hypocrita* and *B. h. sapporoensis* and invasive *B. terrestris* queens mate only once, interspecific mating essentially results in sterility of these queens or at least in a considerable reduction in their fecundity, due to the inviability of the hybrids. For *B. h. sapporoensis* queens, mass escape of commercial *B. terrestris* is basically synonymous with the release of sterile males, as was performed during the eradication of the melon fly *Bactrocera cucurbitae* from the southern islands of Japan with the mass release of flies that were artificially sterilized using γ irradiation [8]. 

To investigate polyandry, the paternity frequency of feral *B. terrestris* on Hokkaido was estimated using only workers’ genotypes and ranged from 1 to 8 with a mean ± standard error of 2.42 ± 0.54 [48] (Table S1). If the effective number is more conservatively estimated from the genotypes of queens and workers, the value ranges from 1 to 4 with a mean of 1.26 ± 0.16. These two values are still exceptionally high among species in the subgenus *Bombus* and when compared with other populations of *B. terrestris*. Unexpectedly, 55.6% (10/18) of the colonies included in the former estimate and 54.5% (6/11) in the latter, were headed by polyandrous queens, suggesting that an increase in mating frequency has occurred in feral colonies of *B. terrestris* on Hokkaido. Of course, caution is necessary when interpreting the effective number of matings, as these values may also be influenced by queen takeover, worker drift, egg dumping and thelytokous parthenogenesis [49]. Our recent study revealed positive associations between mating frequencies of *B. h. sapporoensis* queens and densities of *B. terrestris* colonies: queens of *B. h. sapporoensis* were polyandrous (effective number of matings = 3.61 ± 0.47) where *B. terrestris* colonies were generally more abundant but monandrous (effective number of matings = 1) in areas where they were less abundant (Tsuchida et al. unpublished work). The distance between two populations was about 180 km. These estimated values for the former population were obtained genetically using 18 reared colonies headed by a queen collected in the field. Therefore, worker drifting and egg dumping were ruled out. The polyandrous area overlapped with the polyandrous area of the previous study [48] and the monandrous area was also monandrous for *B. terrestris*. Of course, caution is required because these associations are not causal; however, these lines of evidence do not contradict our hypothesis that both bumblebee species might counteract the strength of RI from their counter-partners.

Although uncertainty still remains, these findings suggest that both bumblebee species might increase their mating frequency when experiencing RI from each other. Polyandry could be an adaptive trait for invasive population because a single queen can carry more patrilines than monandry, which likely decreases the opportunity of diploid male production [50]. In general, multiple mating imposes extra costs on females and these costs can explain the predominance of monandry in bumblebees, as shown in Table S1. Although RI may at present select for polyandrous queens, monandrous queens will again become more adaptive when new reproductive barriers are constructed between the pairs of subspecies or species in the future. Finer-tuned perception of differences in chemical and/or visual cues will be favoured by selection [51]. 

## 7. Biogeographic Issues for Bumblebees in Japan

In 2006, Japan designated *B. terrestris* as an invasive alien species under the Invasive Alien Species Act. After enactment of this law, the number of *B. ignitus* colonies used for pollination increased, as farmers did not require permission to use them [52]. The use of this species throughout Japan could lead to problems because it is not indigenous to Hokkaido, which is biogeographically separated from the other Japanese islands by the Blakiston Line (Figure 1). If *B. ignitus* is introduced onto Hokkaido, new RI could emerge among the three species. Another concern is an issue associated with disease spread by the *Nosema* microsporidian parasite, which could readily horizontally infest other species. Fortunately, the main bumblebee retailers in Japan have not yet sold *B. ignitus* colonies on Hokkaido due to self-management. 

The relationship between *B. ignitus* and *B. h. sapporoensis* is further complicated because both species are also indigenous to Korea and China. *B. ignitus* populations in China, Korea and Japan have diverged due to recent bottlenecks and geographic isolation [53]. In particular, Japanese *B. ignitus* is markedly differentiated from continental populations [53,54] and it is necessary to avoid homogenizing these separate gene pools when using *B. ignitus* as a pollinator. In addition, Korean populations of *B. ignitus* are moderately differentiated within Korea [55] and the same may be true for *B. h. sapporoensis* in Korea and China. If these two species are mass-reared by bumblebee companies and transported among Japan, Korea and China, the native gene pools will be at risk of homogenization.

## 8. Candidate Control Methods against *B. terrestris*

In theory, introduced pollinators can sometimes help native endangered vegetation and the impact on flora of introduced pollinators is not always obvious [26]. *Bombus terrestris* is now widely distributed on Hokkaido and important natural vegetation in Hokkaido may be suffering as a result of this invasive species; in particular, it is found in an alpine area (Daisetsu National Park) in central Hokkaido and in a coastal area (Notsuke-Furen Natural Park) in eastern Hokkaido [56]. The invasion in the Daisetsu area is at a very early stage [57] and the population density remains low. On the other hand, *B. terrestris* is not widely distributed in Honshu, presumably because the climatic conditions in Honshu are hotter and wetter than in Hokkaido, where such conditions might prevent the spread of this species on Honshu. Indeed, analysis of habitat suitability modelling revealed that the susceptibility of invasion by *B. terrestris* is moderate in northern Honshu, while that in southern Honshu is minimal [58]. Similar results were obtained through MaxEnt analysis [59]. Therefore, we should concentrate our attention on controlling the invasive population of this species on Hokkaido, particularly around the two areas with valuable vegetation, Daisetsu and Notsuke-Furen (Figure 1). In other lowland areas of Hokkaido, particularly around open agricultural fields, the density should be higher; suppression in these areas would be more expensive and is not cost-effective at present.

We propose two candidate suppression methods against *B. terrestris* in the two areas: first, eradication through mass release of males of native *B. h. sapporoensis*, so that RI would be induced between the two species; and second, direct application of insecticide against the foraging workers of *B. terrestris*.

During the mass release of males of native species into a population that is genetically structured, the release of males with uniform genetic backgrounds should be avoided to maintain variation in the wild population. Fortunately, this concern is minimal, as no clear genetic differentiation was detected among the populations of *B. h. sapporoensis* on Hokkaido using mtDNA haplotypes and microsatellite markers [60]. However, it is difficult to match the timing of interspecific mating in the field. Therefore, RI through male release is possible but is not a reasonable method.

The second method is application of insecticide against feral nests through foraging workers of *B. terrestris*. Insect growth regulator (IGR) insecticides can effectively suppress the development of eggs, larvae and pupae but do not immediately kill adults. Toxic baits containing IGR have been developed to control the invasive hornet *Vespa velutina nigrithorax* in Japan; the foraging workers carry the bait into their nest and feed it to their larvae. As a result, the larvae die by inhibition of ecdysis [61]. Such toxic bait carried by workers has also been successful for controlling several local populations of invasive ants [62,63,64]. Bumblebees cannot carry such bait because they are pollinators; therefore, we are testing the direct spraying of an insecticide onto the bodies of foraging workers. In preliminary studies, sprayed workers of *B. terrestris* caused the collapse of natal colonies under sub-open conditions (Goka, personal communication). This method is more realistic than male mass release. 

The Plant Protection Act, which aims to protect agricultural plants from pests, has been in force in Japan since 1950, with minor revisions. Article 1 of the act states that “The purpose of this act is to quarantine imported and export plants and domestic plants and to control animals and plants injurious to plants and to prevent them from spreading and thereby ensure the safety and promotion of agricultural production.” Here, injurious animals are defined as follows: “Injurious animals as used in this act mean insects, arthropods such as mites, invertebrates such as nematodes or vertebrates that are injurious to useful plants.” According to this act, the introduction of bumblebees is legal because bumblebees do not appear to injure any agricultural plants. One reason for endorsing the Invasive Alien Species Act is the need to counter the growing threat of accidental release of imported pet animals. However, in 1999, the Plant Protection Act was relaxed, allowing importation of 64 alien stag beetles of interest to many amateur insect collectors and enthusiasts in Japan. Anecdotal evidence suggests that some alien stag beetles have been naturalized in Japan and genetic amalgamation likely occurred with native stag beetles after the introduction of closely related alien beetles. None of the beetles has any apparent injurious traits against agricultural plants. Such relaxation has had many unrecognized negative ecological impacts on native fauna and flora. We should be cognizant that eradication is more costly than introduction and a period of deliberation should occur prior to species introductions, even when the focal animal appears to be useful to farmers and appealing to enthusiasts at the time of introduction.

## 9. Conclusions

We reviewed hybrid production between pairs of subspecies or species of bumblebees in Japan, as a characteristic of RI. Introduced *B. terrestris* and native Japanese bumblebees showed clear RI through inviable egg production. This interference is likely to cause the extinction of native bumblebee species. Unexpectedly, the mating frequency of introduced *B. terrestris* in Hokkaido may have increased. We note that within about 20 years of the introduction of *B. terrestris* to Japan, *B. terrestris* might become polyandrous, most likely to circumvent the direct negative impacts of RI, even though they are essentially a monandrous species. The same process of polyandry evolution could be true for the native bumblebee *B. h. sapporoensis* in Hokkaido. Further studies are needed to confirm the evolution of polyandry in bumblebee species.

Chemical control through spraying IGR against foraging workers is the optimal method for suppressing invasive *B. terrestris*, especially in the two important areas of Daisetsu National Park and Notsuke-Furen Natural Park. This method could be effective in areas with valuable vegetation, where more human power (e.g., civil volunteers) can be invested to control invasive bumblebees than in open agricultural areas.

## Figures and Tables

**Table 1:** Hatchability of eggs derived from intraspecific and interspecific mating.

Mating Pairs	No. of Pairs	No. of Eggs	No. of Hatched Larvae	Hatchability (%)
*Bt* queen × *Bt* male	10	121	93	76.8^a^
*Bi* queen × *Bi* male	45	798	701	87.8^a^
*Bi* queen × *Bt* male	49	566	22	3.9^b^

Bt, Bombus terrestris; Bi, Bombus ignites.

**Table 2:** Sex of eggs and larvae for each colony headed by a single queen, as revealed by microsatellite genotyping.

	No. of Eggs			No. of Larva	
No. of Colonies	Haploid	Diploid	No. of Colonies	Haploid	Diploid
*B. ignitus* gyne × *B. terrestris* male					
15	4	24	11	15	0
*B. terrestris* gyne × *B. ignitus* male					
1	1	4	1	4	0

**Table 3:** The results of sequence analysis for spermatozoa stored in the spermathecae of Japanese native queens.

	Paternity of Spermatozoa Stored in Spermathecae of Queens								
	*B. terrestris*		B. hypocrita*		*B. ignitus*		*B. ardens ardens*		Total
Queen Species	*N*	*freq.*	*N*	*Freq.*	*N*	*freq.*	*N*	*Freq.*	*N*
*B. hypocrita* total	74	0.263	205	0.730	1	0.004	1	0.004	281
*B. h. sapporoensis*	52	0.302	120	0.698	-	-	-	-	172
*B. h. hypocrita*	22	0.202	85	0.780	1	0.009	1	0.009	109
*B. ignitus*	0	0	0	0	43	1.000	0	0	43

Asterisk indicates *B. h. sapporoensis* or *B. h. hypocrita* whose habitats geographically separated into Hokkaido and other main islands. *B. ignitus* and *B. a. ardens* do not distribute in Island of Hokkaido. Reproduced from Kondo et al. (2009) [11].

## Supplementary Materials

The following are available online at https://www.mdpi.com/2075-4450/10/2/59/s1, Table S1: Effective number of queen mating frequency for each bumblebee species.
