# Supplementary material for: Reproductive Interference in an Introduced Bumblebee: Polyandry may Mitigate Negative Reproductive Impact
Source: Insects. 2019 Feb 22;10(2):59. doi: 10.3390/insects10020059 (PMC6409605; doi:10.3390/insects10020059)
Supplement: Supplementary file 1 [file insects-10-00059-s001.pdf]

Table S1. Effective number of queen mating frequency for each bumblebee species.

Subgeneus	Species name	No. of colonies	Effective number of mating#	Polyandrous queen/sampled queen	Reference
Bombias	*Bombus auricomus*	1	1		Payne *et al*. (2003)
Bombus	*B. affinis*	1	1		Payne *et al*. (2003)
	B. florilegus	14	1		Takahashi *et al*. (2008a)
	B. ignitus	7	1		Takahashi *et al*. (2008b)
	B. lucorum	20	1		Estoup *et al*. (1995b)
		12	1		Schmid-Hemple & Schmid-Hemple (2000)
	B. terrestris	45	1		Estoup *et al*. (1995b)
		17	1		Schmid-Hemple & Schmid-Hemple (2000)
		18	2.42	10/18	Inoue *et al*. (2012)
Fervidobombus	*B. fervidus*	1	1		Payne *et al*. (2003)
Megabombus	*B. diversus*	3	1		Kokuvo *et al*. (2009)
	B. hortorum	5	1		Schmid-Hemple & Schmid-Hemple (2000)
Melanobombus	*B. lapidarius*	20	1		Estoup *et al.* (1995b)
		11	1		Schmid-Hemple & Schmid-Hemple

					(2000)
	B. sicheli	2	1		Schmid-Hemple & Schmid-Hemple (2000)
Pyrobombus	*B. ardens*	5	1		Kokuvo *et al*. (2009)
	B. bimaculatus	4	1.06	1/4	Payne *et al.* (2003)
	B. hypnorum	54	-----	16/54	Estoup *et al*. (1995b)
		17	1.13	4/17	Schmid-Hemple & Schmid-Hemple (2000)
		14	1.26	7/14	Paxton *et al*. (2001)
		10	1		Brown *et al*. (2003)
	B. impatiens	10	-----	2/10	Cnaani *et al*. (2002)
		11	1.07	1/9	Payne *et al*. (2003)
	B. mixtus	1	3.57	1/1	Payne *et al*. (2003)
	B. pratorum	35	1		Estoup *et al*. (1995b)
		5	1		Schmid-Hemple & Schmid-Hemple (2000)
	B. ternarius	1	2.04	1/1	Payne *et al*. (2003)
	B. vagans	4	1		Payne *et al*. (2003)
	B. wilmattae	9	1.16	2/9	Huth-Schwarz *et al*. (2001)
Separatobombus	*B. griseocollis*	1	1		Payne *et al*. (2003)

Thoracobombus	*B. honshuensis*	1	1			Kokuvo *et al*. (2009)
	B. pascuorum	6	1			Schmid-Hemple & Schmid-Hemple (2000)
*Psithyrus**	*B. citrinus*	2	2.13		2/2	Payne *et al*. (2003)
	B. insularis	3	1			Payne *et al*. (2003)

*: social parasite

#: effective number of mating calculated by one of the following methods of Starr (1984), Pamilo (1993), and Nilesen et al. (2003), all based on harmonic mean.
